# A Sharp Rise in Autoimmune Encephalitis in the COVID-19 Era: A Case Series

**DOI:** 10.7759/cureus.34658

**Published:** 2023-02-05

**Authors:** Pardis Saffari, Raya Aliakbar, Argin Haritounian, Rafik Mughnetsyan, Catherine Do, Jamie Jacobs, Julia Hoffer, Robert Arieli, Antonio K Liu

**Affiliations:** 1 Internal Medicine, Adventist Health White Memorial, Los Angeles, USA; 2 Neurology, Adventist Health White Memorial, Los Angeles, USA; 3 Neurology, Loma Linda University School of Medicine, Loma Linda, USA

**Keywords:** molecular mimicry, voltage-gated potassium channel autoimmune encephalitis, anti-glutamic acid decarboxylase, autoimmune encephalitis, covid

## Abstract

Background: Autoimmune encephalitis was very rare prior to the current pandemic. A sharp rise in cases has been observed from March to August of 2022 in Los Angeles. Such an increase, especially with certain types of antibodies, may point toward the possibility of post-infectious autoimmune encephalitis. While review articles on autoimmune encephalitis during this pandemic have been published, a sharp rise in one geographic area within a short period of time has not been documented yet.

Aims: To report an alarming increase in autoimmune encephalitis with mostly positive glutamic acid decarboxylase (GAD) and/or voltage-gated potassium channel (VGKC) antibodies over six months during 2022 in Downtown Los Angeles.

Material and methods: This is an observational case series from one neurocritical care practice in Downtown Los Angeles. Autoimmune encephalitis antibody panels were sent to patients with altered mental status or neurologic deficits of unclear etiology from March to August of 2022.

Results: Of the 29 patients tested, 12 reports came back positive. Ten had positive GAD and/or VGKC antibodies, one had a positive myelin oligodendrocyte glycoprotein antibody, and one had a positive leucine-rich glioma-inactivated 1 protein antibody; a 41% positive rate.

Conclusions: This observation has important implications: (1) We may be entering an era of heightened autoimmune encephalitis. (2) These occurrences may be post-infectious in nature at this point of the pandemic. (3) Mostly GAD and VGKC antibodies have been identified (10 of them), which may point toward a new direction of research from a molecular mimicry standpoint. (4) To benefit patients, clinicians need to be aware of such disease manifestations and increase testing; resources must be increased to improve test availability and shorten turnaround time; and treatment, which is expansive, must be made widely available for these potentially reversible diseases.

## Introduction

We report a case series of 12 patients who presented with neurologic symptoms and positive autoimmune encephalitis antibody titers in Los Angeles at two community teaching hospitals over five months. Most patients had confirmed COVID-19 infection, exposure, and/or had previously been vaccinated.

Long-term effects have been variable for those who contracted COVID-19, including pulmonary fibrosis, hypercoagulopathy, and neurologic symptoms. A paper published by Chou et al. in May 2021 showed that neurological manifestations were found in approximately 80% of patients hospitalized with COVID-19. The most common self-reported symptoms included headache (37%) and anosmia or ageusia (26%), whereas the most common neurological signs or syndromes were acute encephalopathy (49%), coma (17%), and stroke (6%). The presence of clinically captured neurologic signs or syndromes was associated with an increased risk of in-hospital death [1].

Central nervous system (CNS) manifestations of COVID-19 were hypothesized to result from virus access through angiotensin converting enzyme (ACE) receptors, passing through the blood-brain barrier and causing inflammation or death of neurons. Furthermore, the effects of the virus on the CNS are not limited to those caused by direct entry. COVID-19 infection causes a systemic hyperinflammatory cascade or “cytokine storm” that damages neurons. Damage to the CNS is also reported through an immune-mediated CNS response observed in patients with less severe forms of the disease [2].

Autoimmune encephalitis is rare; the incidence rate from 2006 to 2015 was estimated to be 1.2 cases per 100,000 people [3]. The prevalence of specific neural autoantibodies was as follows: myelin oligodendrocyte glycoprotein (MOG), 1.9/100,000; glutamic acid decarboxylase (GAD), 1.9/100,000; unclassified neural autoantibody, 1.4/100,000; leucine-rich glioma inactivated 1 (LGI1), 0.7/100,000; collapsin response mediator protein 5, 0.7/100,000; N-methyl-D-aspartate (NMDA) receptor, 0.6/100,000; antineuronal nuclear antibody type 2, 0.6/100,000; and glial fibrillary acidic protein α, 0.6/100,000 [3]. Voltage-gated potassium channel (VGKC) antibody incidence is not well established.

Despite the low prevalence of autoimmune encephalopathies, 12 cases have been recognized in our hospitals with positive autoimmune antibodies over the past five months. This is a significant rise in the prevalence of such a rare disease. The patients presented with a variety of neurologic symptoms.

## Materials and methods

This is an observational study aimed at understanding the nature of an alarming surge in the number of patients presenting with altered mental status (AMS) and negative regular neurological workup who were eventually diagnosed with autoimmune encephalitis. Loma Linda University Institutional Review Board has granted an exemption notice (IRB# 5220253) for this study. Written consents were obtained from patients or their next of kin. Between the months of March and August 2022, 29 patients had presented with AMS and/or other neurological conditions whose diagnosis remained unclear after neurological consultation and regular workup; the Autoimmune Neurologic Diseases Reflexive Panel, Serum (ARUP 3004070) offered by the Associated Regional and University Pathologists, Inc (ARUP) was sent to all 29 patients [4]. This test consists of 16 different antibodies. Twelve of them came back positive. Chart review was performed on these 12 patients and their profiles were analyzed. The main limitation of this test is that the average turnaround time is 20 days. Therefore, treatment was usually initiated prior to confirmation of positive antibodies based on clinical suspicion, symptoms, and lack of other etiologies. Moreover, these observations were made from only one neurocritical care practice and were probably below the true incidence during this period of time in this geographic area. 

## Results

Case 1

**Figure 1:**
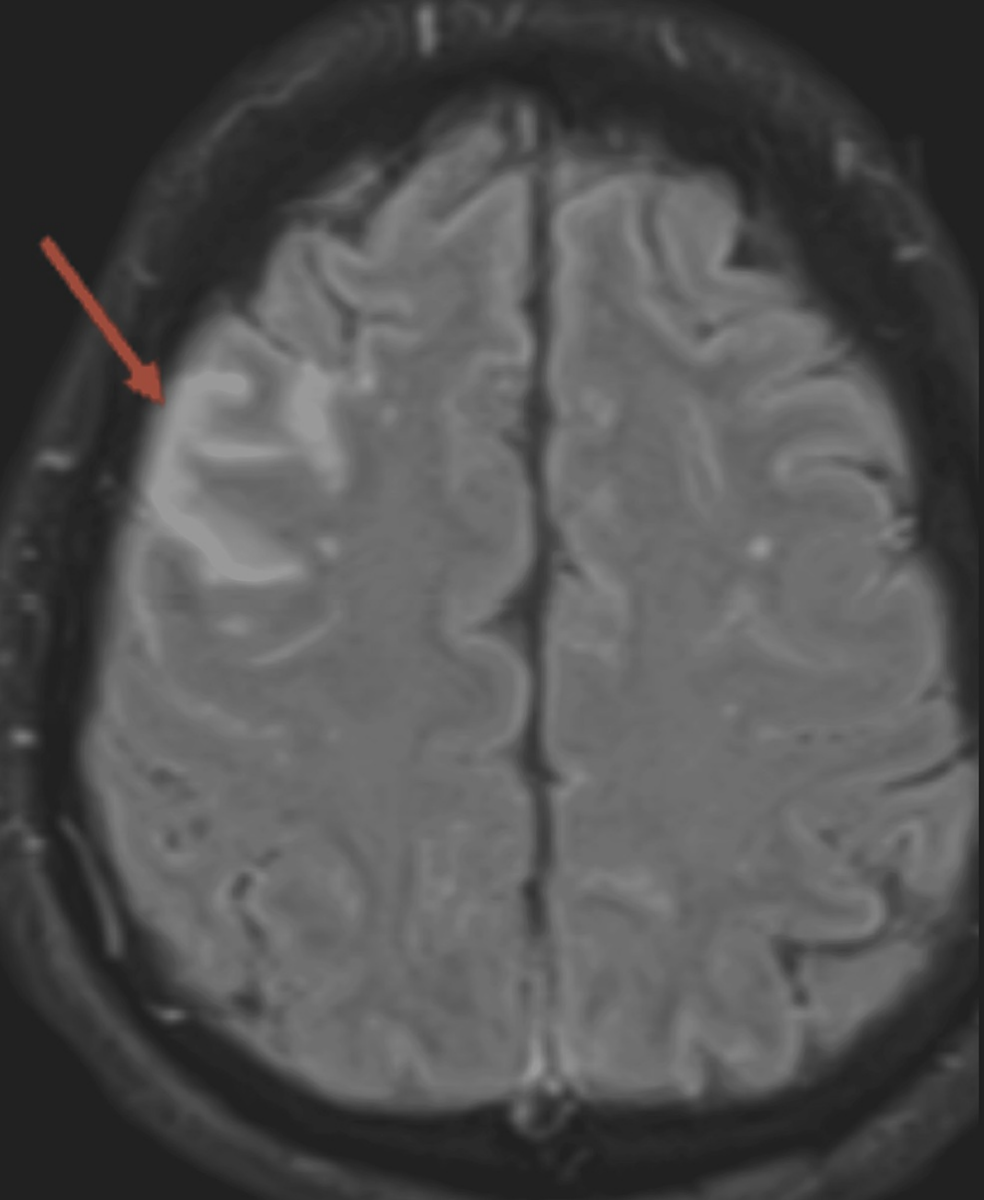
Brain MRI showing curvilinear FLAIR signal abnormality in gyriform pattern in the right frontal lobe in a patient with GAD and VGKC antibodies (red arrow). FLAIR: fluid-attenuated inversion recovery; GAD: glutamic acid decarboxylase; VGKC: voltage-gated potassium channel.

Patient 1 was a 53-year-old previously healthy man who presented with AMS. He contracted the COVID-19 infection one month prior to presentation. His COVID infection ran a mild course with no respiratory symptoms. He was oriented only to name. He was agitated, combative, and violent without focal neurologic deficits. Brain MRI showed fluid-attenuated inversion recovery (FLAIR) abnormality in the right frontal lobe (Figure 1). Cerebrospinal fluid (CSF) analysis was relatively benign (Table 1). An extensive infectious workup (including infectious encephalitis) was negative. He had elevated titers of GAD and VGKC antibodies. The patient received a course of plasmapheresis followed by intravenous immunoglobulin (IVIG) and high-dose methylprednisolone. Therapy continued with rituximab 375 mg/m^2^. Despite aggressive treatment, the patient had minimal improvement through the 40-day hospitalization. He remained oriented to name and was intermittently able to follow simple commands. He was bedridden and nonfunctional and was eventually placed in a skilled nursing facility.

**Table 1:** Characteristics of 12 patients with autoimmune encephalitis. AMS: altered mental status; CPM: central pontine myelinolysis; DWI: diffusion-weighted imaging; FLAIR: fluid-attenuated inversion recovery; GAD: glutamic acid decarboxylase; IVIG: intravenous immunoglobulin; LGI1: leucine-rich, glioma inactivated 1; MBP: myelin basic protein; MIS-A: multisystem inflammatory syndrome in adult; MOG: myelin oligodendrocyte glycoprotein; PCR: polymerase chain reaction; SLE: systemic lupus erythematosus; VGKC: voltage-gated potassium channel; VDRL: Venereal Disease Research Laboratory; GM2: ganglioside-monosialic acid 2; GD1b: ganglioside-disialosyl 1b; EBV: Epstein-Barr virus; dsDNA: double-strand DNA.

	Case 1	Case 2	Case 3	Case 4	Case 5	Case 6	Case 7	Case 8	Case 9	Case 10	Case 11	Case 12
Age	53	57	21	56	64	49	57	59	30	65	58	48
Sex	Male	Female	Female	Male	Male	Female	Male	Female	Male	Male	Female	Male
COVID history	+Nasal swab PCR one month prior	No known infection	+Nasal swab PCR three weeks prior. Severe MIS-A	No known infection	Never confirmed, had mild flu	No known infection	No known infection	+Nasal swab PCR on admission	No known infection	No known infection	No known infection	No known infection
COVID vaccination	None	Moderna secondshot 14 days prior	None	Pfizer x 2, last dose 10 months prior	Moderna second shot six months prior	Moderna second shot 12 months prior	Moderna second shot 12 months prior	Moderna second shot 12 months prior	None	Pfizer x 3. Last dose five months prior	Unknown	Unknown
COVID risk factor	Not known	None	Crowded living situation with continuous sick contact	Works in ski resort. Constant contact with no mask	Close contact with COVID+ family members three weeks prior	Unclear	A resident of a skilled nursing facility	Crowded living situation with sick contacts	Lived with a sister who was COVID+ three weeks prior	Lived with a grandson who was COVID+ one month prior	Close contact with COVID+ family members four weeks prior	Homeless. Lived on the street and in various shelters
Significant history	None	None	Possible SLE	Had CPM with Na 99 mmol/L, alcoholic	Highly functional, work	HIV+, neurosyphilis, substance abuse	DM, HTN, acute renal failure, wheelchair bound but normal mentation	Obese	Substance abuse	None	Substance abuse	Homeless
Symptoms	Incoherent, disoriented, agitated, combative, strong, bedridden	Dopa-responsive dystonia and chorea. No AMS	Malaise, seizure, AMS, generalized weakness	AMS, lock-in syndrome	Incoherent, disoriented, memory loss, aggressive, and psychosis. No weakness	Rapid deterioration, from normal to coma over two weeks	Progressed from normal to coma over two months	Disoriented, incoherent, and obtunded	Baclofen-responsive chorea and ataxia	AMS	Coma	Incoherent, agitated. Strong, combative. Refused care. Paced hallways.
Autoimmune encephalitis antibodies	GAD 148.8 IU/ml, VGKC 44 pmol/L	GAD >250 IU/mL, VGKC 104 pmol/L	GAD 213.4 IU/mL, VGKC 154 pmol/L	GAD 39.5 IU/mL	LGI1 Ab IgG 1:320	VGKC 230 pmol/L	VGKC 64 pmol/L	VGKC 94 pmol/L	GAD >250 IU/ml, VGKC 59 pmol/L	MOG 1:20	GAD 48.8 IU/mL, VGKC 42 pmol/L	VGKC 96 pmol/L
CSF analysis	Clear. WBC 7/mm^3^, protein 32 mg/dL	Clear. WBC 4/mm^3^, protein 193 mg/dL	Clear. WBC 13 /mm^3^, protein 36 mg/dL	Clear. WBC 0/mm^3^, protein 61mg/dL, MBP 106 ng/mL	Clear. WBC 2/mm^3^, protein 53 mg/dL	Clear. WBC 2/mm^3^, protein 54 mg/dL, VDRL not reactive	Clear. WBC 10/mm^3^, protein 165 mg/dL, MBP 9.1 ng/mL	Clear. WBC 0/mm^3^, protein 300 mg/dL	Clear. WBC 0/mm^3^, protein 38 mg/dL	Clear. WBC 0/mm^3^, protein 53 mg/dL	Clear. WBC 0/mm^3^, protein 103 mg/dL	Not done. Refused
Other labs	Unrevealing	Unrevealing	Positive ANA, dsDNA, EBV IgM, Salmonella, warm hemolytic anemia	GM2 antibody elevated	Unrevealing	HIV CD4 330	Unrevealing	Unrevealing	GD1b antibody elevated	Unrevealing	Unrevealing	Unrevealing
Brain MRI	Abnormal FLAIR, Figure 1	Negative	Negative	Consistent with CPM, Figure 2	Negative	Negative	Extensive white matter disease, Figure 3	Negative	Negative	Multiple ring enhancement, Figure 4	Abnormal DWI, Figure 5	Abnormal DWI, Figure 6
Treatment	Plasmapheresis, steroid, rituximab	Plasmapheresis, steroid, rituximab, ropinirole	Steroid, seizure medication	IVIG, steroid, rituximab	IVIG, steroid, rituximab	IVIG, steroid, rituximab	IVIG, steroid, rituximab	IVIG, steroid	Baclofen, IVIG, rituximab, cyclophosphamide	IVIG, steroid	IVIG, steroid	Refused
Outcome	Moderate encephalopathy, bedridden with tracheostomy and feeding tube	Improved, ambulate with a walker	Recovered, went home	Moderate encephalopathy, bedridden with tracheostomy and feeding tube. Quadriplegic	Psychosis resolved. Mild confusion. Went home	Slightly better. Severe encephalopathy	Much improved. Basic orientation follows	Improving, oriented, malaise persisted	Partially improved, then relapsed. Improved again after retreatment. Now walking	Mentation better. MRI-enhancing lesions resolved	Steadily improved	Left hospital prior to lab became available

Case 2

Patient 2 was a 57-year-old previously healthy female. She received her second Moderna booster 14 days prior to presentation. Five days prior to presentation, she developed involuntary body movements. The movements were a combination of myoclonus, dystonia, and chorea. It progressively worsened, and she was bedridden, dysarthric, dysphagic, and unable to use her limbs functionally. A trial of various medications led to the discovery that her symptoms were responsive to dopaminergic agents. She remained oriented and coherent throughout. An MRI of the brain showed no significant findings. CSF analysis showed elevated protein. She tested positive for GAD and VGKC antibodies (Table 1). Her symptoms improved after plasmapheresis and a course of rituximab 375 mg/m^2^. By the time she was discharged home, she was able to feed herself, use the bathroom with minimal assistance, and ambulate with a walker. She was discharged on low-dose ropinirole.

Case 3

Patient 3 was a 21-year-old female with COVID-19 infection three weeks prior to presentation. The classic COVID-19 symptoms ran a relatively benign course. However, prior to admission, she developed malaise, generalized weakness, AMS, and seizures. Her hospital course was complicated by multisystem inflammatory syndrome in adults (MIS-A). She was initially altered, but her mentation steadily improved and returned to baseline by the end of the first week. There was no seizure recurrence. MRI, CSF analysis, and EEG were all unrevealing. She tested positive for GAD and VGKC antibodies (Table 1). The patient returned to her baseline neurologically without the need for immune modulatory therapy.

Case 4

**Figure 2:**
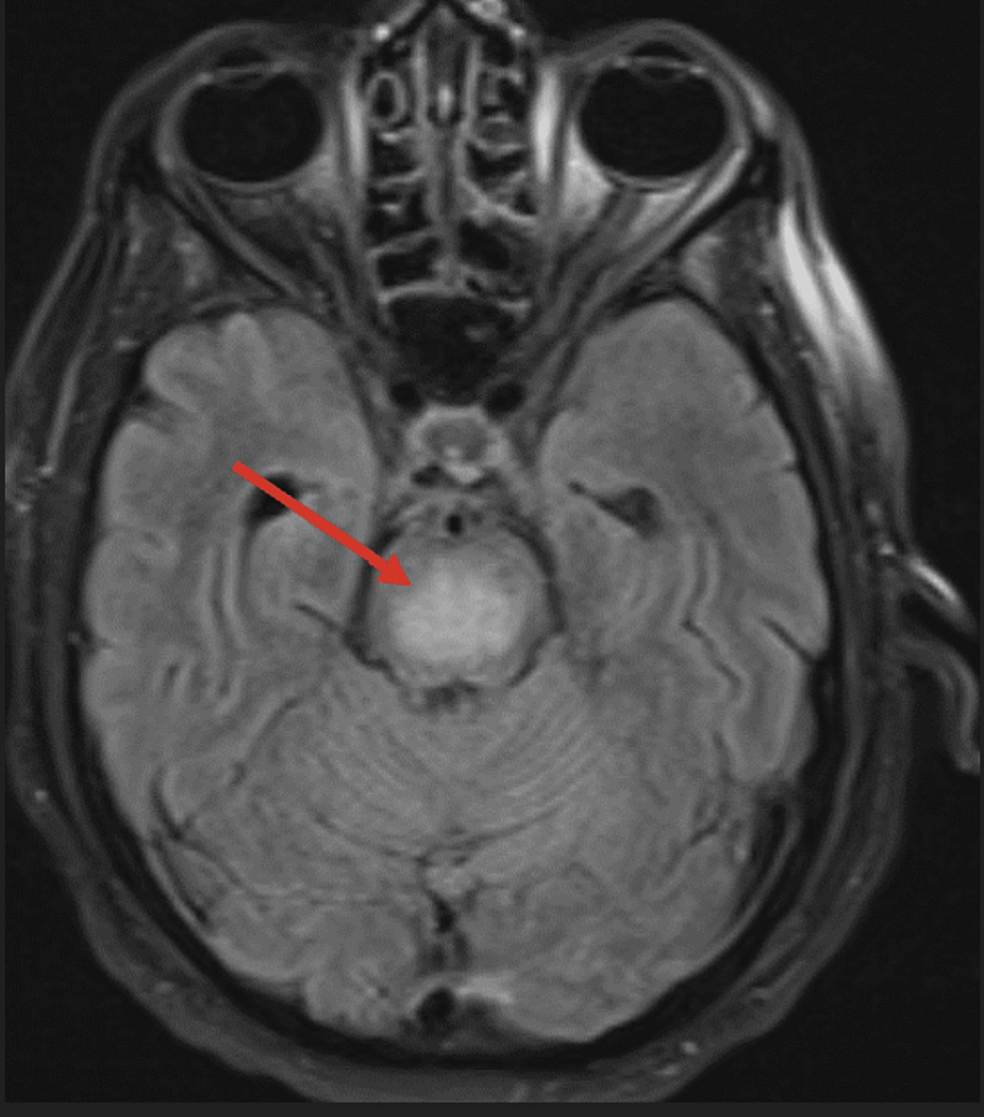
Brain MRI FLAIR sequence showing CPM in a patient with GAD antibody (red arrow). CPM: central pontine myelinolysis; FLAIR: fluid-attenuated inversion recovery; GAD: glutamic acid decarboxylase.

Patient 4 was a 56-year-old alcoholic male who presented to our emergency department for AMS and weakness. Two weeks prior, he was admitted to the intensive care unit at an outside hospital for hyponatremia of 99 mmol/L. It was corrected by more than 10 mmol/L/day. He was sent home but developed signs and symptoms of central pontine myelinolysis (CPM) a few days later. He had no confirmed COVID-19 infection and his last COVID-19 vaccination was nine months prior. He worked at an out-of-state ski resort with many sick contacts and no mask requirement. MRI findings were consistent with CPM (Figure 2). His symptoms progressed to a locked-in syndrome. He tested negative for COVID-19 nasal swab polymerase chain reaction (PCR) but positive for GAD antibody (Table 1). IVIG and rituximab 375 mg/m^2^ were administered. After three weeks of hospitalization, the patient’s neurologic symptoms improved. He was able to mouth words, recognize family, and follow simple commands. Despite developing spastic contracture, he could move his upper extremities with 2/5 strength.

Case 5

Patient 5 was a 64-year-old previously healthy male. He had close contact with multiple COVID-19-positive family members three weeks prior to the onset of AMS. He had mild flu-like symptoms, but COVID-19 diagnosis was never confirmed. The patient presented with memory loss, incoherent speech, disorientation, psychosis, and agitation. MRI of the brain, EEG, and CSF analysis was all unremarkable. He had elevated titers of LGI1 antibody to 1:320 (Table 1). The patient improved after IVIG, rituximab 375 mg/m^2^, and methylprednisolone treatment. On discharge, he was alert and oriented with no focal neurologic deficits. However, his family reported that he was slower in response and not at his “intellectual self.”

Case 6

Patient 6 was a 49-year-old HIV-positive female. She was employed and independent. Her COVID-19 infection history was unclear. She had received Moderna vaccinations one year prior. She visited the emergency department for AMS. She continued to deteriorate over two weeks and became comatose. Her MRI and EEG were negative. CSF analysis showed a normal cell count and a mild protein elevation. She had a history of neurosyphilis, but her current CSF Venereal Disease Research Laboratory (VDRL) was negative. Her CD4 count was 330 cells/mm^3^. A significant elevation of VGKC autoantibody to 230 pmol/L was detected (Table 1). IVIG, methylprednisolone, and rituximab 375 mg/m^2^ were administered. After 75 days of hospitalization, she recovered somewhat and was alert and oriented to name and place. She was able to carry on a simple conversation with family on FaceTime. However, she remained impulsive with intermittent anger outbursts, which was uncharacteristic of her prior to presentation.

Case 7

**Figure 3:**
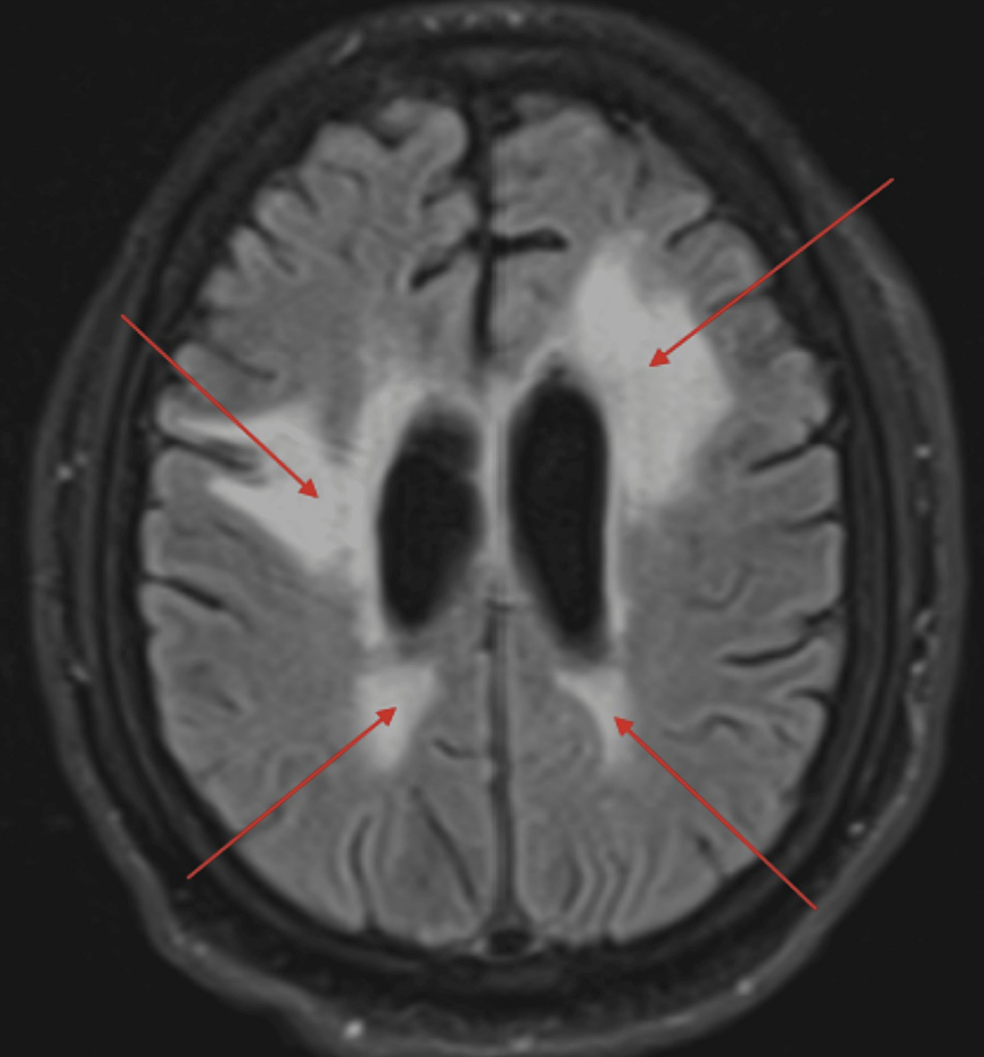
Extensive white matter disease in a patient with VGKC antibody (red arrows) on MRI FLAIR sequence. VGKC: voltage-gated potassium channel; FLAIR: fluid-attenuated inversion recovery.

Patient 7 was a 57-year-old male nursing home resident who presented to the hospital with two months of progressive AMS. Despite being physically disabled, he was mentally intact as per his family. By the time of admission, he was completely unresponsive. He has no proven COVID-19 infection, but there were multiple exposures at the nursing home. His CSF analysis showed WBC of 10/mm^3^, protein of 165 mg/dL, and positive myelin basic protein. MRI of the brain showed extensive white matter disease and, incidentally, a small subacute (2 mm) infarct at the left cerebellum (Figure 3). He was positive for VGKC antibody (Table 1). After a course of IVIG, rituximab 375 mg/m^2^, and methylprednisolone, he recuperated over six weeks to the point of being oriented to self and place. He could name family members, follow commands, and lift both upper extremities against gravity.

Case 8

Patient 8 was a 59-year-old obese, previously healthy female with a progressive AMS. She was bedridden, incoherent, disoriented, and agitated. MRI, CT, and CT angiogram of the brain were negative. EEG repeatedly showed slowing without epileptic discharges. CSF analysis showed 0/mm^3^ WBC and protein of 300 mg/dL. She was positive for COVID-19 nasal swab PCR. Her viral illness ran a mild course without respiratory difficulty. She tested positive for VGKC antibody at 94 pmol/L (Table 1). She received a course of IVIG and methylprednisolone. Her symptoms improved over six weeks; she became oriented and was able to ambulate with minimal assistance. However, she still had a short-term memory deficit by the time of her transfer to a skilled nursing facility.

Case 9

Patient 9 was a 30-year-old man with a history of substance abuse who presented with involuntary chorea and muscle spasm. He lived with his sister, who was ill with COVID-19 three weeks prior to presentation. He tested negative for COVID without prior immunization. On exam, the patient appeared to be coherent and oriented with mostly upper extremities chorea, dystonia, and involuntary movement. Motor strength was 5/5 in all four extremities. He had spastic gait and dysdiadochokinesia. His chorea was baclofen responsive. His symptoms progressed, and he became bedridden. The patient was started on IVIG and methylprednisolone, and symptoms improved. However, he experienced a relapse one month later, and labs showed elevated titers: ganglioside-disialosyl 1b (GD1b) of 55, GAD > 250 IU/mL, and VGKC 59 pmol/L (Table 1). A second round of IVIG was started in addition to rituximab and cyclophosphamide. The patient’s symptoms improved, and he was able to ambulate with minimal assistance by the time of discharge.

Case 10

**Figure 4:**
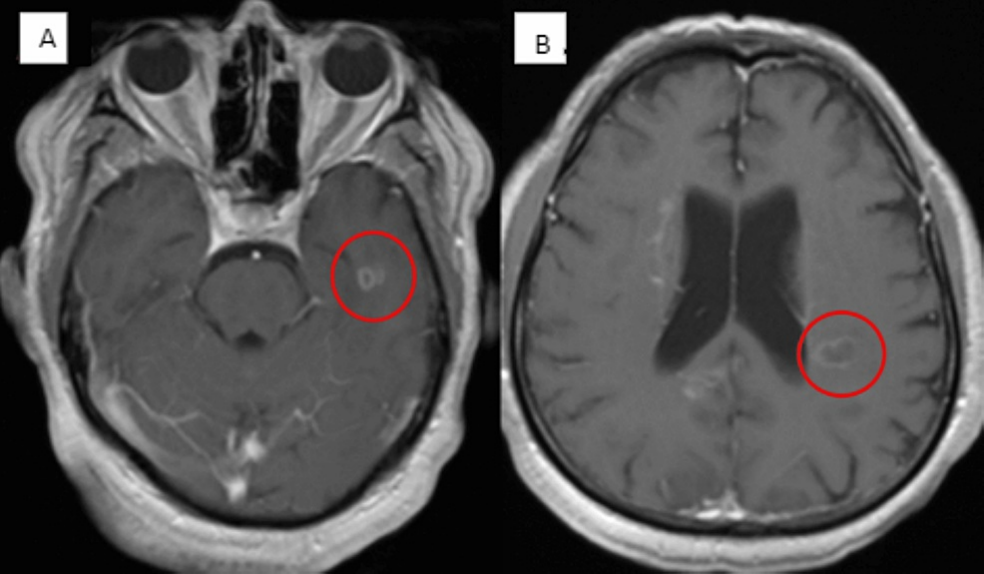
Brain MRI showing ring-enhancing lesions on T1 in the left temporal lobe (A) and left periventricular area (B) in a patient with a positive MOG antibody. MOG: myelin oligodendrocyte glycoprotein.

Patient 10 was a 65-year-old man who was previously healthy and functional. He experienced gradual deterioration in his mentation and function over eight weeks. He received his third Pfizer vaccine four months prior. He was exposed to his COVID-19-positive grandson one month prior, but he was never tested. He had received a negative workup for his AMS from an outside hospital. Three weeks passed without improvement, and upon admission to our hospital, he was generally oriented and non-focal. The family reported he was inappropriate, disinhibited, and could not perform daily activities. Brain MRI showed ring-enhancing lesions (Figure 4). Spinal MRI was negative. Infectious, immunosuppressed state, and malignancy workup were all negative. HIV was negative, and CSF showed 0/mm^3^ WBC and protein of 53 mg/dL. The patient decided against further invasive workup and was discharged to outpatient care. He received a course of methylprednisolone. Two weeks after discharge, MOG antibody IgG titer came back positive at 1:20 (reference is <1:10). He was brought back to the hospital for further immune modulation treatment. He has experienced slow but steady improvement since.

Case 11

**Figure 5:**
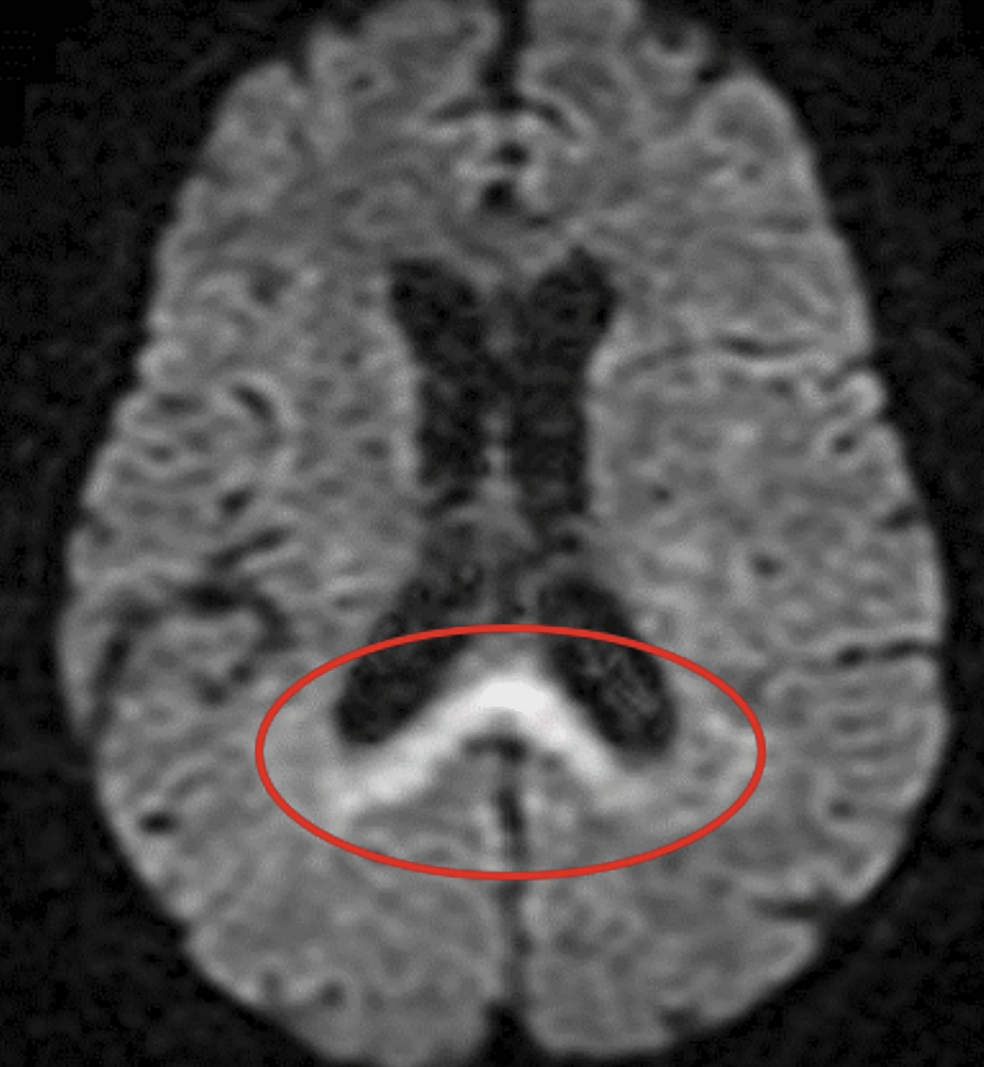
Brain MRI showing an abnormal DWI signal in the posterior corpus callosum in a patient with a positive GAD antibody (red circle). DWI: diffusion-weighted imaging; GAD: glutamic acid decarboxylase.

Patient 11 was a 58-year-old female who was previously functional but had a history of substance abuse. She had no confirmed COVID-19 infection but was living with a COVID-19-positive individual one month prior to presentation. She was brought to the hospital after being found down for an unknown period of time. She was found to have respiratory failure and was intubated. She progressed from obtundation to coma over four days. Her MRI showed an abnormal diffusion-weighted imaging (DWI) signal at the posterior corpus callosum (Figure 5). Her CSF showed 0/mm^3^ WBC and protein of 103 mg/dL. With no other etiologies identified, IVIG and methylprednisolone were initiated. Eventually, GAD and VGKC came back positive at 48.9 IU/mL and 42 pmol/L. The patient improved after treatment. After four weeks of hospitalization, she was oriented to name and place and follow commands. 

Case 12

**Figure 6:**
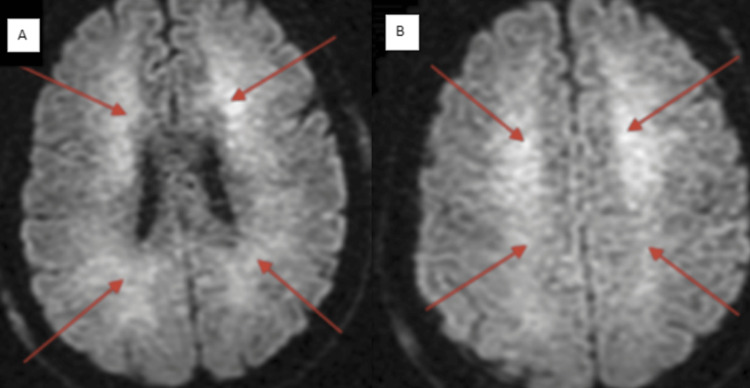
Brain MRI showing a diffuse abnormal DWI signal in a subcortical manner (A) and (B) in a patient with a positive GAD antibody (red arrows). DWI: diffusion-weighted imaging; GAD: glutamic acid decarboxylase.

Patient 12 was a 48-year-old homeless male who could not provide any history. His baseline mentation was unknown, and he lived on the street and in various shelters. Throughout his hospital stay, he repeatedly refused care and management. Therefore, we did not perform CSF analysis. He was never obtunded but agitated, combative, and incoherent. He was physically strong and would pace the hallways for long periods. His brain MRI showed extensive subcortical DWI abnormalities that did not follow vascular distribution (Figure 6). His lab came back positive for VGKC at 96 pmol/L. He refused medications. He was placed in a shelter and lost to follow-up.

The 12 cases described here presented with neurological symptoms between March and August 2022 in Downtown Los Angeles. With 12 cases being positive out of 29 total cases tested, the positive rate is an alarming 41%. After analyzing these 12 patients, we found out they either tested positive for COVID-19, were vaccinated, or had been exposed prior to presentation. Symptomatically, 10 out of 12 patients had varying degrees of AMS, from mild encephalopathy to unresponsiveness. One patient had coincidental CPM. The remaining two patients had hyperkinetic movement disorders, and their mental status changes were mild. Their movement disorder was similar to the hyperkinetic subtype of von Economo disease after the 1918 Spanish flu [5]. 

Antibody-wise, there were interesting findings. ARUP Laboratories runs the Autoimmune Neurological Diseases Reflexive Panel on 16 different antibodies, but only four were positive in our observations. One patient had a positive LGI1, and another patient had a positive MOG antibody; but they both presented with AMS. The remaining 10 patients were all positive for GAD and/or VGKC. There were nine positive VGKC and six positive GAD antibodies in total. One patient is GAD positive only and four patients were VGKC positive only. The remaining five patients were positive for both GAD and VGKC. Eight had AMS, while the two movement disorder predominant patients were positive for both GAD and VGKC. Interestingly, ganglioside-monosialic acid 2 (GM2) and GD1b, both ganglioside antibodies, were positive in two patients. GM1 was positive in the patient with CPM, and GD1b was positive in one of the patients with chorea. 

MRI findings were interesting for six patients, ranging from DWI abnormality to FLAIR sequence signal alteration. One patient had enhancing lesions. MRI was normal in the remaining six patients. No consistent pattern was identified. Eleven out of 12 patients had CSF analysis. Only one patient had a mildly elevated WBC count. Eight out of 11 patients had elevated CSF protein. Such findings usually point away from infectious etiologies.

Treatment was directed at clearing the serum and dampening the immune response through IVIG, methylprednisolone, rituximab, and cyclophosphamide. There were mixed responses. All made some improvement, with a few patients close to recovery. 

## Discussion

We are more than two years into the current COVID-19 pandemic already. Most, if not all, of our patients had some degree of exposure to the virus and/or had been vaccinated. They all presented with AMS or some other neurological manifestation but their diagnosis remained unclear after the initial neurological workup. Clinical suspicion led to the testing for autoimmune encephalitis. Treatment was usually initiated before laboratory confirmation was made available. Twelve out of 29 patients eventually tested positive with a positive rate of 41%. While various types of autoimmune encephalitis have been described during this pandemic [6,7], a sharp increase in any particular type of antibody in a single geographic area over a short period has not been described in the literature. The degree of increase is astonishing, given that each of the antibodies has an incidence of less than 10 per 100,000. A series of positive tests within six months in one area may imply that the incidence is thousands of times higher than usual. It implies that we may be entering an era of heightened autoimmune encephalitis.

Clinically, the symptoms themselves were not novel. Antibodies directed against the VGKC have been demonstrated to be involved in several CNS and PNS autoimmune diseases. They are characterized by a wide range of neurological complications, including epilepsy, limbic encephalitis, and acquired neuromyotonia [8,9]. In our cases, patients with elevated VGKC titers had a range of symptoms, including seizures, altered mental status, obtundation, chorea, and dystonia. GAD antibody has been associated with motor symptoms, balance disorders, and various cognitive impairments such as hallucinations, aggression, and memory loss [10-12]. Our patients had similar symptoms, such as involuntary movements and chorea (baclofen- or dopaminergic-responsive), muscle spasms, and gait difficulty. LGI1 antibody encephalitis has been associated with seizures, memory loss, and sleep disorders [13]. MOG antibody is usually associated with neuromyelitis optica spectrum disorder and postinfectious acute disseminated encephalomyelitis. Encephalitis has been described but is less common. Ring-enhancing MRI lesions are rare [14]. 

In the sequela of the COVID-19 pandemic, there is much interest in the long-term effects of the virus on the body. In this paper, we present twelve cases of rare neurological diseases with elevated autoimmune antibody titers. It is beyond the scope of this paper to prove that these neurological cases were for sure postinfectious in nature. More research is needed to determine if COVID-19 infection and/or vaccination cause cytokine production of varying degrees that result in a heightened inflammatory and immune response; this heightened response may be a “catalyst” to many other neuropathological processes by activating autoimmunity. This idea is in line with growing data on the enhanced immune response in the pathophysiology of neurological disorders after COVID-19 [6]. However, if this is the only mechanism, one should expect an increase in all antibodies. Since GAD and VGKC antibodies made up most of the positive antibodies, molecular mimicry is a more likely factor. Could GAD and VGKC antibodies be the explanation for the surge in autoimmune encephalitis during the current pandemic? Furthermore, the two patients who presented with movement disorder had positive GAD and VGKC antibodies. Could they be the key to the mechanism of von Economo disease [15]? 

## Conclusions

Autoimmune encephalitis is a serious but treatable condition with recovery potential; thus, the current observation of an alarming surge in autoimmune encephalitis with mostly GAD and/or VGKC antibodies detected during the current COVID-19 pandemic has important implications. Clinicians should have increased suspicion of autoimmune-related neurological pathologies in the COVID era. As such, we should order autoimmune encephalitis antibodies when deemed appropriate and initiate treatment without waiting for the titer results. More specifically, clinicians should increase their surveillance of GAD or VKGC markers and other autoimmune diseases, as this may be a significant sequela of this pandemic. Laboratories should make these tests more readily available, and the turnaround time should be shorter. Practitioners and hospitals should anticipate an increase in the use of immune modulation therapies. More studies are needed to work out the pathophysiology and molecular aspects of our current observation, as well as to study the efficacies of various immune modulations.
